# Sepsis-induced long-term immune paralysis – results of a descriptive, explorative study

**DOI:** 10.1186/s13054-016-1233-5

**Published:** 2016-02-29

**Authors:** C. Arens, S. A. Bajwa, C. Koch, B. H. Siegler, E. Schneck, A. Hecker, S. Weiterer, C. Lichtenstern, M. A. Weigand, F. Uhle

**Affiliations:** Department of Anesthesiology, Heidelberg University Hospital, Im Neuenheimer Feld 110, 69120 Heidelberg, Germany; Department of Anesthesiology and Intensive Care Medicine, University Hospital of Giessen and Marburg, Giessen, Germany; Department of General and Thoracic Surgery, University Hospital of Giessen and Marburg, Giessen, Germany

**Keywords:** Sepsis, Immunology, Immune system, Immunocompromised

## Abstract

**Background:**

Long-lasting impairment of the immune system is believed to be the underlying reason for delayed deaths after surviving sepsis. We tested the hypothesis of persisting changes to the immune system in survivors of sepsis for the first time.

**Methods:**

In our prospective, cross-sectional pilot study, eight former patients who survived catecholamine-dependent sepsis and eight control individuals matched for age, sex, diabetes and renal insufficiency were enrolled. Each participant completed a questionnaire concerning morbidities, medications and infection history. Peripheral blood was collected for determination of i) immune cell subsets (CD4^+^, CD8^+^ T cells; CD25^+^ CD127^-^ regulatory T cells; CD14^+^ monocytes), ii) cell surface receptor expression (PD-1, BTLA, TLR2, TLR4, TLR5, Dectin-1, PD-1 L), iii) HLA-DR expression, and iv) cytokine secretion (IL-6, IL10, TNF-α, IFN-γ) of whole blood stimulated with either α-CD3/28, LPS or zymosan.

**Results:**

After surviving sepsis, former patients presented with increased numbers of clinical apparent infections, including those typically associated with an impaired immune system. Standard inflammatory markers indicated a low-level inflammatory situation in former sepsis patients. CD8^+^ cell surface receptor as well as monocytic HLA-DR density measurements showed no major differences between the groups, while CD4^+^ T cells tended towards two opposed mechanisms of negative immune cell regulation via PD-1 and BTLA. Moreover, the post-sepsis group showed alterations in monocyte surface expression of distinct pattern recognition receptors; most pronouncedly seen in a decrease of TLR5 expression. Cytokine secretion in response to important activators of both the innate (LPS, zymosan) and the adaptive immune system (α-CD3/28) seemed to be weakened in former septic patients.

**Conclusions:**

Cytokine secretion as a reaction to different activators of the immune system seemed to be comprehensively impaired in survivors of sepsis. Among others, this could be based on trends in the downregulation of distinct cell surface receptors. Based on our results, the conduct of larger validation studies seems feasible, aiming to characterize alterations and to find potential therapeutic targets to engage.

## Background

Sepsis remains a big challenge in modern intensive care medicine and is still a leading cause of death [1–3]. It is an interwoven immune reaction to distinct microorganisms, which comprises systemic inflammatory response syndrome (SIRS) with a “cytokine storm” accompanied by the counteracting, so-called compensatory anti-inflammatory response syndrome (CARS) [4, 5].

In the case of overwhelming CARS, the phenomenon of immune paralysis occurs due to immune cell apoptosis and functional impairment of lymphocytes and phagocytes, also associated with increased anti-inflammatory and decreased pro-inflammatory cytokine production [6–9]. Overshooting of the anti-inflammatory response predisposes the host to secondary bacterial infection, infection with opportunistic microorganisms and reactivation of latent viruses [7, 10–13]. In contrast to the initial causative insult of sepsis, which can be treated by source control and anti-infective therapy, no therapeutic or preventive strategies are established to combat the deleterious effects of immune paralysis during the period of CARS [14]. Furthermore, the immune cell phenotype of patients who die from sepsis has features consistent with immunosuppression [15]. Patients with impaired immune function are prone to incomplete recovery from sepsis. They often present with typical syndromes that follow the SIRS-CARS reaction: persistent inflammation-immunosuppression catabolism syndrome (PICS) and multiple organ dysfunction syndrome (MODS) [16]. Besides sophisticated intensive care treatment, a relevant number of survivors of the initial septic event ultimately fall victim to PICS or MODS in the long-term.

Even after complete recovery many patients who recover from sepsis have an impaired quality of life for years and are found to have increased mortality [17–19]. Studies have illustrated ongoing mortality of up to 43 % after one year [17], 44.9 % after two years [20] and 74.2 % after five years beyond hospital discharge [21]. Sepsis-related long-lasting impairment of the immune system is believed to be the underlying reason for these delayed deaths in those who initial survive sepsis. However, studies investigating the immune status of survivors of sepsis in humans are missing. In this prospective, cross-sectional pilot study we aimed to explore for the first time the phenomenon of sepsis-induced long-term immune paralysis (SLIP) in a limited number of patients who survived catecholamine-dependent sepsis.

## Methods

### Enrollment of patients who had previously survived sepsis

The local ethics committee at the Medical Faculty of the Justus-Liebig-University Gießen, Germany approved the study protocol (trial code: 291/13). We identified all patients who had been diagnosed with sepsis (ICD10: A41.X) and who had required vasopressors or inotropes (catecholamine-dependent sepsis), and had been treated on the interdisciplinary surgical intensive care unit at Gießen University Hospital between January 2011 and December 2013. Data were extracted from our patient data management system ICUData (IMESO® GmbH, Giessen, Germany). All patients identified were contacted by mail. After evaluation of possible exclusion criteria, written informed consent was collected and individuals were enrolled in the study. Exclusion criteria were: onset of sepsis less than nine months or more than 60 months prior to the date of investigation, pregnancy, participating in another interventional study, chronic viral infections (HIV, hepatitis), end-stage renal failure, autoimmune diseases and patients taking high-dose corticosteroids (hydrocortisone >200 mg/day or equivalent) or other immunosuppressive medications.

Eight individuals who had never suffered from sepsis were matched for age, sex, diabetes and renal insufficiency and checked for the presence of exclusion criteria, and were subsequently enrolled as controls after giving written informed consent. Blood (50 mL) was collected in heparinized, ethylenediaminetetraacetic acid (EDTA) or serum tubes by peripheral venipuncture from each study participant and immediately processed.

### Questionnaire

All study participants were asked to complete a 63-item questionnaire on current and past morbidities, current medications and clinically apparent infections during the last twelve months. In addition, former data on the sepsis-associated hospital stay were extracted from our patient data management system (focus of infection and time since sepsis).

### Standard laboratory parameters

For standard laboratory tests, blood samples were processed in the routine hospital laboratory. Blood count (white blood cells, red blood cells, hemoglobin, hematocrit, mean corpuscular volume, mean corpuscular hemoglobin, mean corpuscular hemoglobin concentration and platelets) and additional parameters of relevance in the context of infections (fibrinogen, aspartate transaminase, alanine transaminase, γ-glutamyl transferase, C-reactive protein (CRP) and procalcitonin) were measured according to in-house standards.

### Characterization of immune cell subsets

To assess the number of T cell subsets and monocytes, whole blood was stained and measured by flow cytometry as described in detail below. The percentage of CD4^+^ and CD8^+^ T cells was calculated as the fraction of all CD3^+^ events, while the amount of regulatory T cells (CD4^+^ CD25^+^ CD127^-^) was calculated as the fraction of all CD4^+^ events. Last, the percentage of monocytes was calculated as the amount of CD14^+^ cells from all measured cellular events.

### Quantitative HLA-DR analysis on monocytes

Expression of human leucocyte antigen-antigen D-related (HLA-DR) on CD14^+^ monocytes was measured by flow cytometry according to the manufacturer’s recommendations (Quantibrite HLA-DR/Monocyte antibody cocktail, BD Bioscience, Heidelberg, Germany). Briefly, 50 μL of EDTA-anticoagulated whole blood was incubated with 20 μl of the antibody cocktail and incubated for 30 min. Afterwards, erythrocyte lysis was performed by adding 450 μl FACS Lysing solution (BD Bioscience, Heidelberg, Germany) and further incubation for 15 minutes. Measurement was immediately done on a FACSCalibur flow cytometer (BD Bioscience). In addition, a 4-point calibration curve (Quantibrite PE Beads, BD Bioscience) was also measured daily to enable the transformation of the measured sample values to molecules of HLA-DR.

### Expression of cell surface proteins

Cell surface expression analysis was performed by flow cytometry. For each target of interest, 100 μL whole blood was incubated with the corresponding antibodies and incubated for 30 minutes at 4 °C, followed by a lysing step of 15 minutes at room temperature after addition of 2 mL fluorescence-activated cell sorting (FACS) lysing solution. Finally, the cells were washed twice (1.000 rpm, 5 minutes, 4 °C) with cold phosphate-buffered saline (PBS; Thermo Fisher Scientific, Waltham, MA, USA) containing 1 % bovine serum albumin (fraction V, protease-free; Carl Roth, Karlsruhe, Germany).

The following antibodies were used for the identification of immune cell subsets: fluorescein isothiocyanate (FITC)-CD3 (BD Bioscience, #555916), FITC-/phycoerythrin (PE)-CD4 (BD Bioscience, #555346/555347), PE-CD8 (BD Bioscience, #555367), FITC-CD14 (BD Bioscience #555397), PE-CD25 (BD Bioscience, #555432), Alexa Fluor 647-CD127 (BD Bioscience, #558598). For the quantitative measurement of expression levels on T cells, the following antibodies were used: programmed cell death 1 (PD-1) (Biolegend, San Diego, USA, #329908), B- and T-lymphocyte attenuator (BTLA) (Biolegend, #344510), or cytotoxic T-lymphocyte-associated protein 4 (CTLA-4) (Biolegend, #349908) (all conjugated to allophycocyanin (APC)). For the quantitative measurement of expression levels on monocytes, the following antibodies were used in addition: toll-like receptor (TLR)2 (Biolegend, #309708), TLR4 (Biolegend, #312806), TLR5 (Abcam, Cambridge, UK, #4.5119), Dectin-1 (Biolegend, #355404) or programmed cell death ligand 1 (PD-1 L) (Biolegend, #329706) (all conjugated to PE). Furthermore, for every antibody used, an according sample was stained with the same amount of isotype-matched non-binding antibody (isotype control). Percentages of positive cells represent the fraction of cells with fluorescence above isotype control.

To obtain robust and reliable quantitative results without bias due to the day-to-day variance of the instrument, we measured a 4-point calibration curve for both PE and APC (QUANTUM™ MESF beads, Bangs Laboratories, Fishers, IN, USA). Fluorescence intensity of each sample was normalized by subtracting the staining intensity of the according isotype control. Subsequently, conversion to molecules of equivalent soluble fluorophore (MESF) was done be recalculating the normalized intensity to the results of the calibration curve.

### Cytokine secretion of ex vivo-stimulated whole blood

For the functional assessment of the immune cells, whole blood was diluted 1:1 with RPMI1640 containing proprietary GlutMAX™ (Thermo Fisher Scientific, Waltham, MA, USA) and 5 % fetal bovine serum (Ultra-low endotoxin; Cell Concepts GmbH, Umkirch, Germany) and incubated with α-CD3/28 (eBioscience, Frankfurt, Germany; T cell stimulation), ultrapure lipopolysaccharide (LPS) (0111:B4; TLR4 agonist, activates monocytes and dendritic cells) or depleted zymosan (β-1,3-D-glucan; TLR2/Dectin-1 agonist, activates monocytes and dendritic cells) (both Invivogen, San Diego, CA, USA).

For T cell activation, microplates were coated in advance by incubation of 50 μL α-CD3 antibody solution (10 μg/mL) for 24 h. After incubation, the wells were washed twice with sterile PBS and 300 μL of diluted blood and α-CD28 antibody (2 μg/Ml) were added. LPS and zymosan stimulation was performed directly in the blood by adding the agonists in a final concentration of 100 ng/mL and 25 μg/mL, respectively.

All stimulation was done in triplicate for 24 h. After incubation, the samples were centrifuged (3.000 rpm, 5 minutes) and supernatant recovered. As the readout, the secretion of cytokines (IL-2, IL-4, IL-6, IL-10, IL-17A, TNF and IFN-γ) were measured using a multiplex Th1/Th2/Th17 cytometric bead array (BD Bioscience).

### Statistical analysis

The present pilot study was set up with an estimating intention to assess the variances of the endpoints to enable sample size estimation for further studies. Because of lacking data, no *a priori* sample size calculation was performed for this study. Based on the study intention and the small sample size, no group comparisons were performed. Visualization in scatter plots was done using GraphPad Prism (Version 5.0f, GraphPad Software, La Jolla, CA, USA).

## Results

### Enrollment and specimen collection

Long-term survivors of catecholamine-dependent sepsis were identified (n = 172) and contacted by mail 9–52 months after the septic event. Of all interested responders (n = 14), 8 former patients were eligible to be enrolled in the study. Afterwards, 8 individuals matched for age, sex, diabetes, and renal insufficiency, who had never suffered from sepsis, were enrolled as controls.

The clinical characteristics of the patients who had survived sepsis and the control group are shown in Table 1. In the post-sepsis group, the median period between sepsis and the study time point was 26 (9–52) months. The most frequent focus of infection was urogenital (50 %), followed by necrotizing fasciitis (25 %) and pulmonary or endoprosthesis infection (12.5 % each). Further clinical characteristics (especially age, sex, diabetes and renal insufficiency) were similar in the two groups. However, distinct differences were found in prescribed medications e.g., anticoagulant, blood pressure and diuretic medication.

**Table 1:** Clinical characteristics of controls and patients who survived sepsis

	Control (n = 8)	Sepsis (n = 8)
Age, median (range)	59 (34–85)	60 (36–82
Male sex	5 (62.5)	5 (62.5)
Body mass index, kg/m^2^, median (range)	26.4 (18.1–34.3)	24.4 (22.2–40.4)
Time since sepsis, months, median (range)		26 (9–52)
Infection focus responsible for sepsis		
Urogenital		4 (50)
necrotizing fasciitis		2 (25)
Pulmonary		1 (12.5)
Endoprothesis infection		1 (12.5)
Morbidities (current and past)		
*Heart and circulation*		
Myocardial infarction	2 (25)	2 (25)
Myocarditis	0 (0)	0 (0)
Stroke	1 (12.5)	0 (0)
Pulmonary artery embolism	0 (0)	0 (0)
Thrombosis	0 (0)	0 (0)
*Airway and lung*		
Asthma	0 (0)	0 (0)
chronic bronchitis	0 (0)	2 (25)
Pneumonia	0 (0)	2 (25)
Exacerbation of chronic obstructive pulmonary disease	0 (0)	1 (12.5)
*Kidney*		
Insufficiency	1 (12.5)	1 (12.5)
Pyelonephritis	0 (0)	1 (12.5)
Glomerulonephritis	0 (0)	0 (0)
*Gastrointestinal tract and metabolism*		
Chronic inflammatory bowel disease	0 (0)	0 (0)
Ulcus	0 (0)	1 (12.5)
Gastritis	1 (12.5)	1 (12.5)
Diabetes	3 (37.5)	3 (37.5)
Current medications	6 (75)	7 (87.5)
Anticoagulants	0 (0)	3 (37.5)
Anti-platelet agents	5 (62.5)	3 (37.5)
Antihypertensive medication	2 (25)	5 (62.5)
Diuretics	1 (12.5)	4 (50)
Diabetes medication	3 (37.5)	3 (37.5)
Insulin	2 (25)	3 (37.5)

Absolute number (percentage), if not otherwise specified

### Characteristics of clinically apparent infections during the previous year

Of the sepsis survivors 62.5 % had experienced at least one infection during the previous 12 months (Table 2). One of those patients suffered five episodes of upper airway infection. At least one episode of antibiotic treatment was prescribed to four individuals in the sepsis survivors. Three different types of infection occurred (oral candidiasis, herpes zoster and lower airway infection), which are typically associated with an impaired immune system. None of the patients in the control group was diagnosed with an infection or was treated with anti-infective medication.

**Table 2:** Characteristics of clinically apparent infections during the previous year

	Control (n = 8)	Sepsis (n = 8)
Individuals with ≥1 infection	0 (0)	5 (62.5)
Incidence per annum, number (range)		1 (0–5)
Outpatient		2 (25)
Stationary		2 (25)
Antibiotic therapy		4 (50)
Application of blood components		2 (25)
Site of infection		
Upper airway		3 (37.5)
Lower airway		2 (25)
Urogenital		0 (0)
Gastrointestinal		1 (12.5)
Central nervous		0 (0)
Special infectious entities		
Herpes zoster		1 (12.5)
Herpes simplex		0 (0)
Oral candidiasis		1 (12.5)
Cytomegalovirus (re)infection		0 (0)

Absolute number (percentage), unless specified otherwise

### Exclusion of current acute infections

Standard inflammatory markers were determined to screen for current infectious diseases. White blood cell count was within the physiological range in all but one individual (13.2 cells/μL × 10^3^) in the control group (Table 3). CRP was slightly elevated in eight sepsis survivors and seven control individuals. Despite all values being <50 mg/L (threshold for severe infection), there was a minor trend towards increased CRP levels in patients with a history of sepsis. Procalcitonin (PCT) was marginally increased (0.1; 0.1; 0.2 ng/ml) in three sepsis survivors. Thus, the presence of relevant but not clinically apparent infective disorders at the time of enrollment, which could affect our analysis, was unlikely. However, the slightly elevated CRP and PCT values in sepsis survivors indicate a chronic low-level inflammatory status.

**Table 3:** Blood count and additional laboratory parameters

	Control (n = 8)			Sepsis (n = 8)		
	Median	Perc 05	Perc 95	Median	Perc 05	Perc 95
WBC, cells/μL × 10^3^	5,6	3,8	13,2	7,7	5,3	9,9
Erythrocytes, cells/μL × 10^6^	4,8	3	5,3	4,55	4,1	6,1
Hemoglobin, g/L	140	88	158	137	106	170
Hematocrit, %	0,41	0,26	0,48	0,42	0,35	0,51
MCV, fL	86,5	85	93	89,5	83	96
MCH, pg	29,65	28,7	31,9	28,9	25,8	31,7
MCHC, g/L	340,5	331	353	327,5	306	342
Platelets, cells/μL × 10^3^	268	179	346	261	231	320
Fibrinogen, g/L	3,38	2,54	5,43	3,55	2,29	5,21
AST, U/L	28	16	33	20	11	55
ALT, U/L	25	12	52	22	16	73
GGT, U/L	13	7	81	34	9	155
CRP, mg/L	1,76	0	32,53	10,01	0,88	23,65
PCT, ng/mL	0	0	0	0	0	0,2

*perc* percentile, *WBC* white blood cell count, *MCV* mean corpuscular volume, *MCH* mean corpuscular hemoglobin, *MCHC* mean corpuscular hemoglobin concentration, *AST* aspartate transaminase, *ALT* alanine transaminase, *GGT* γ-glutamyl transferase, *CRP* C-reactive protein, *PCT* procalcitonin

### Characterization of circulating immune cells

**Fig. 1:**
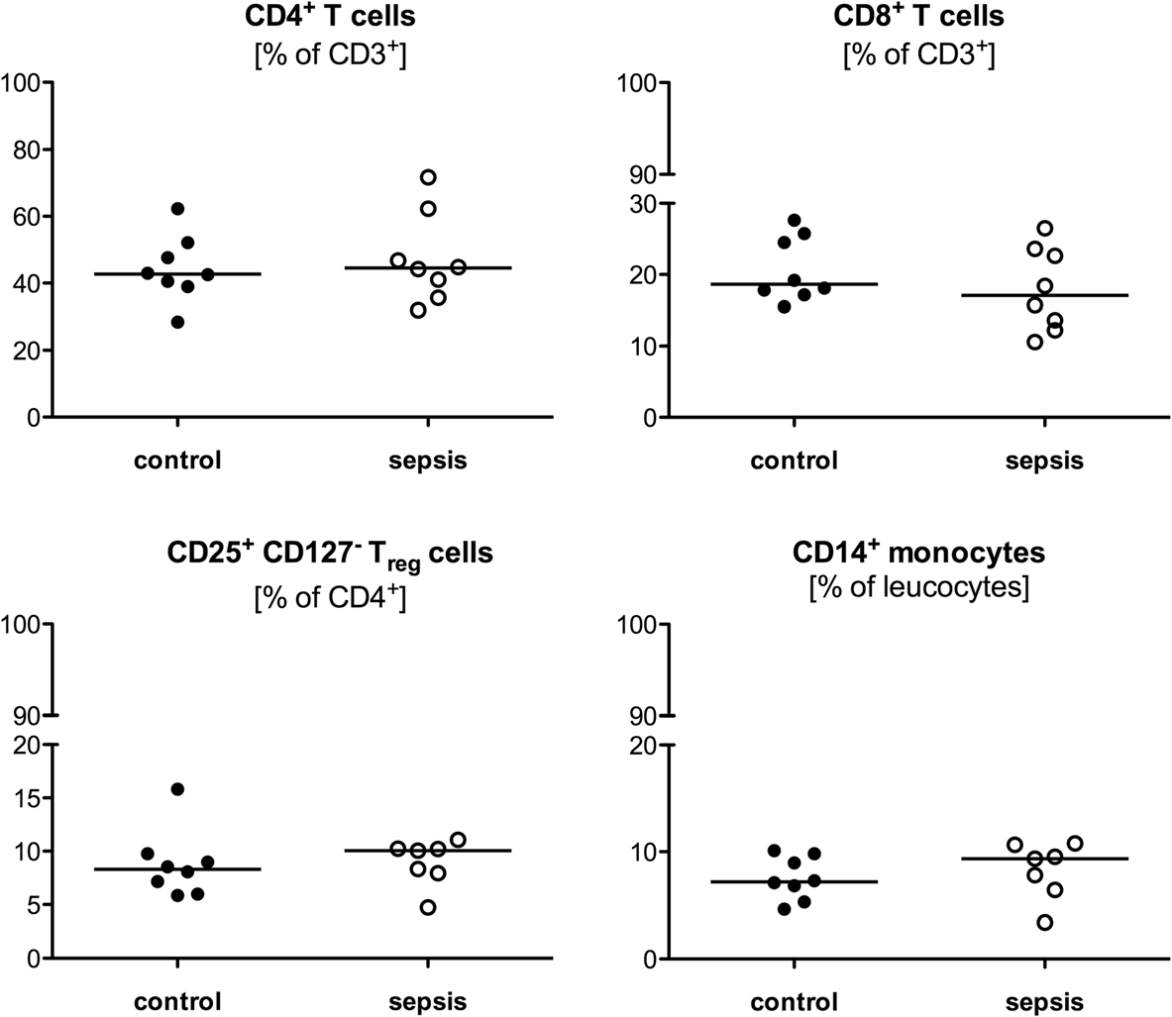
Characterization of immune cells subsets. Peripheral blood was collected from eight survivors of sepsis and matched controls and analyzed by flow cytometry to determine the percentage of circulating CD4+ and CD8+ subsets of CD3^+^ T cells, the amount of regulatory T cells (CD25^+^ CD127^-^ Treg) as a fraction of all CD4^+^ cells, and CD14^+^ monocytes of all leucocytes. The markers used are described in “Methods”. Each data point represents an individual patient. *Horizontal lines* indicate median values

CD4^+^ and CD8^+^ subsets of CD3^+^ T cells were determined by flow cytometry analysis (Fig. 1). Additionally, the percentage of regulatory T cells (CD25^+^ CD127^-^ Tregs) and CD14^+^ (antigen-presenting) monocytes as part of all leukocytes were determined. No substantial differences between the two groups were observed.

**Fig. 2:**
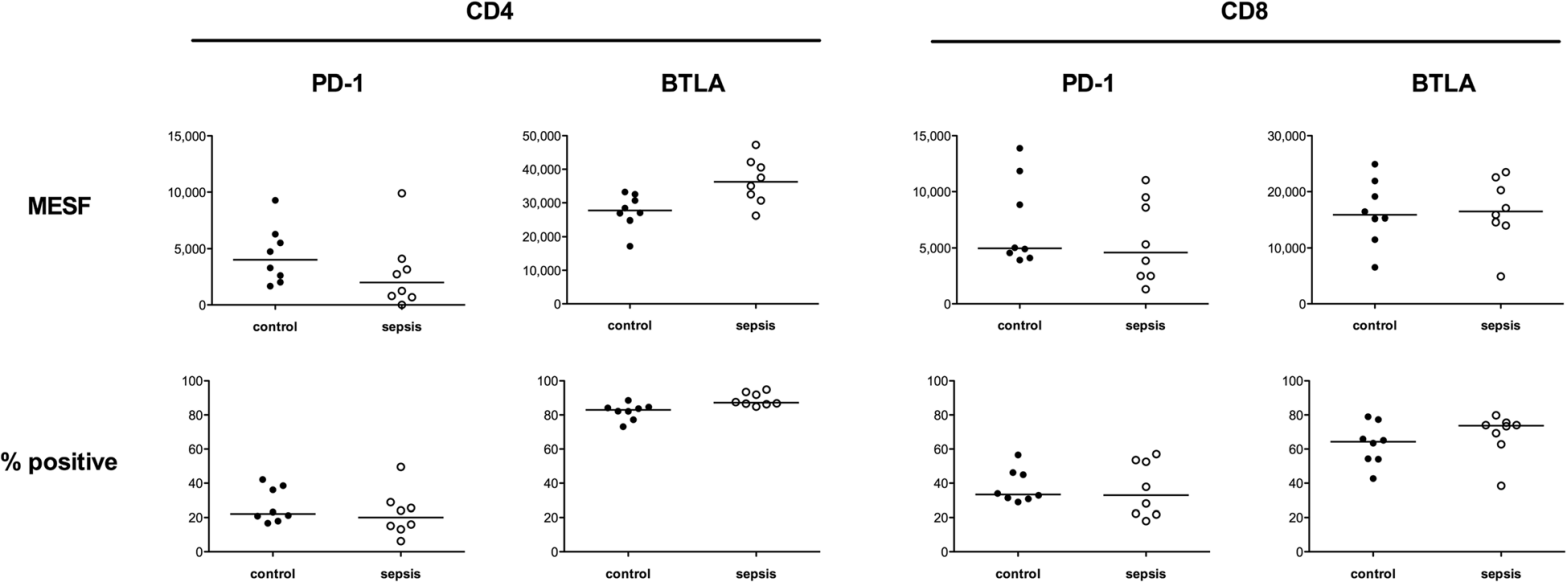
Determination of the pattern of expression of specific receptors on CD4^+^ and CD8^+^ T cells. Peripheral blood was collected from eight survivors of sepsis and matched controls and stained for the indicated markers, followed by determination of percent positive relative to isotype control staining (*bottom line*) and quantification of fluorescence intensity by molecules of equivalent soluble fluorochrome (*MESF*) calculation (*upper line*) as described in “Methods”. Each data point represents an individual patient. *Horizontal lines* indicate median values. *PD* programmed cell death, *BTLA* B- and T-lymphocyte attenuator

Furthermore, we determined expression levels for important negative regulators of lymphocyte function. Therefore we measured PD-1, CTLA-4 and BTLA receptor expression within the CD4^+^ and CD8^+^ T cell subsets by 1) quantifying the percentage of receptor-positive cells and 2) assessing a surrogate parameter of the cell surface receptor density (MESF) (Fig. 2). No expression of CTLA-4 was measurable (data not shown). In CD4^+^ cells of sepsis survivors, the receptor density of PD-1 appeared to be downregulated, but was upregulated for BTLA. In CD8^+^ cells no substantial changes were seen. In summary, this is indicative of two opposing mechanisms of negative immune cell activation feedback in CD4^+^ cells in patients who have survived sepsis.

**Fig. 3:**
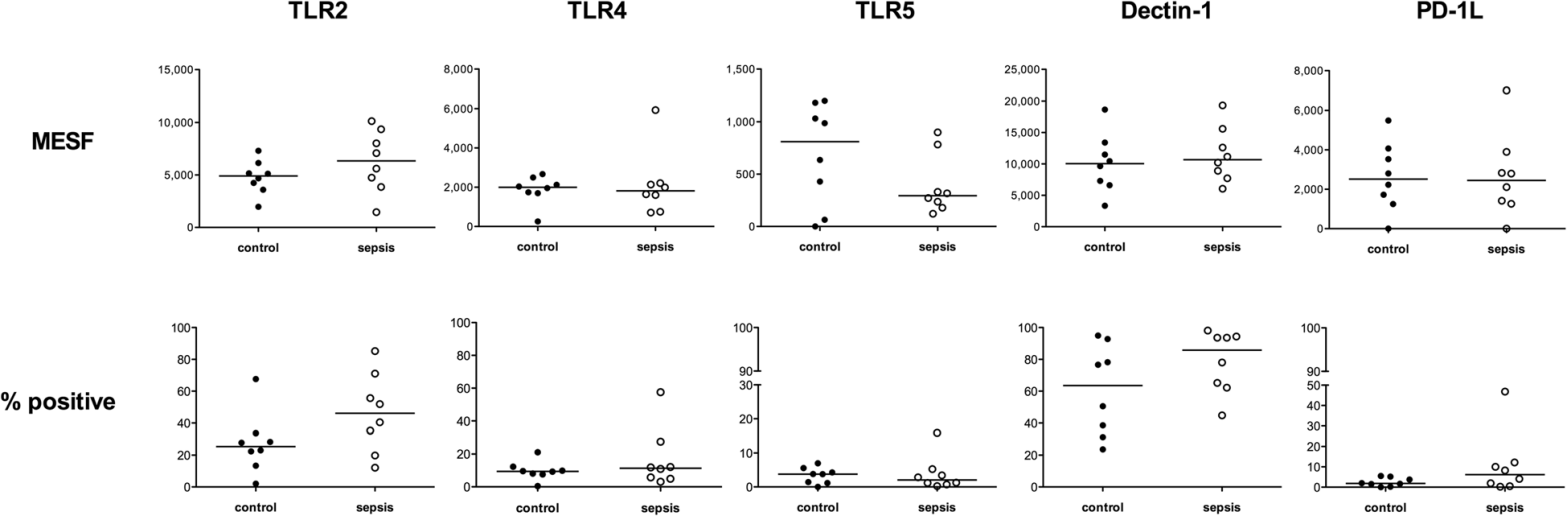
Determination of expression pattern of specific receptors on monocytes. Peripheral blood was collected from eight survivors of sepsis and matched controls and stained for the indicated markers, followed by determination of percent positive relative to isotype control staining (*bottom line*) and quantification of fluorescence intensity by molecules of equivalent soluble fluorochrome (*MESF*) calculation (*upper line*) as described in “Methods”. Each data point represents an individual patient. *Horizontal lines* indicate median values. *TLR* toll-like receptor, *PD-1 L* programmed cell death ligand

Next we sought to clarify whether the initiation of innate immune responses of monocytes via pattern recognition receptors and the negative regulatory molecule PD-1 ligand (PD-1 L) might be disturbed in sepsis survivors due to modified expression levels. Therefore, we determined the surface protein expression of different toll-like-receptors (TLR2, TLR4, TLR5), Dectin-1, and PD-1 L (Fig. 3). For TLR4 and PD-1 L there were no substantial differences between the two groups. Dectin-1-positive cells were more frequent but Dectin-1 density was not affected in the sepsis survivors. There was a slight increase in TLR2^+^ cell count and receptor density. The most notable and coherent effect was seen for TLR5, which is a fundamental player in pathogen-associated molecular pattern recognition (bacterial flagellin) and therefore an activator of innate immunity. Its receptor-positive cell count and especially the receptor density were substantially decreased, which points towards a selectively impaired innate immune system in this special aspect.

**Fig. 4:**
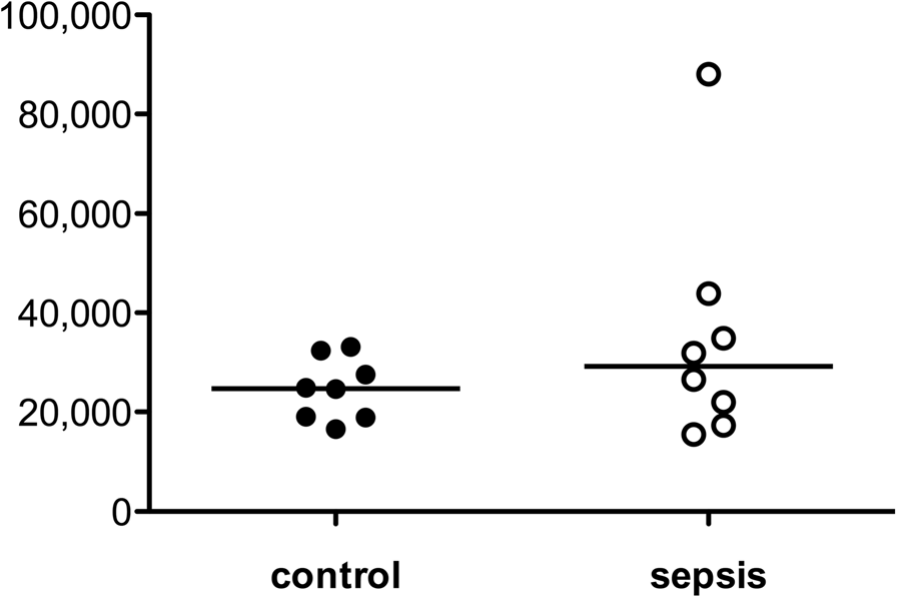
Determination of human leukocyte antigen (HLA)-DR expression on monocytes. Peripheral blood was collected from eight survivors of sepsis and matched controls and HLA-DR expression was measured. Each data point represents an individual patient. Horizontal lines indicate median values

No evident differences were seen in the monocytic HLA-DR receptor density as a surrogate marker of global immune function (Fig. 4).

### Cytokine secretion of ex-vivo-stimulated whole blood

**Fig. 5:**
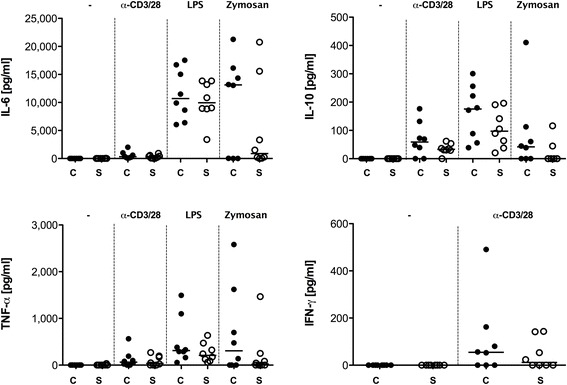
Cytokine secretion of ex-vivo-stimulated whole blood. Peripheral blood was collected from eight survivors of sepsis (*S*) and matched controls (*C*). Whole blood was stimulated with either CD3/28 antibody (α-CD3/28), lipopolysaccharide (LPS) or Zymosan. Supernatants were harvested after 24 h and TNF-α, interferon-γ (*IFN-γ*), and IL-6 and IL-10 were measured by enzyme-linked immunosorbent assay. Each *data point* represents an individual patient. *Horizontal lines* indicate median values. *CD* cluster of differentiation

Last, we determined the immune competence of individual patients to react to distinct inflammatory stimuli. Whole blood from sepsis survivors and controls was either incubated with the T cell activator α-CD3/28, or the monocyte/macrophage-activators LPS (via TLR4) or zymosan (via TLR2/Dectin-1). Subsequently, effects on the expression of the cytokines IL-2, IL-4, IL-6, IL-10, IL-17A, TNF-α and IFN-γ were measured (Fig. 5). No IL-2, IL-4, and IL-17A secretion was detectable (data not shown). For the other parameters determined, the post-sepsis group had decreased cytokine responses. There were pronounced trends towards lower IFN-γ production after stimulation with α-CD3/28 and IL-10 production after LPS activation. Most impressively, stimulation with zymosan, a yeast surface protein, was followed by substantially reduced production of IL-6, IL-10 and TNF-α. The observed responses did not depend on the sole number of leucocytes present in the sample. In fact, only the LPS-induced TNF secretion correlated significantly with the number of leucocytes, but paradoxically the relationship was negative (Spearman *r* –0.5303, *p* = 0.0346; data not shown). All of the abovementioned cytokines were also assessed in native plasma from the participants, to rule out the possibility of their presence as chronic immunogenic stressors, inducing maladaptation of the immune cells. We found no detectable concentration for any cytokine (data not shown).

Taken together, the net effect of detection and reaction to important activators of both the innate and the adaptive immune system seems to be impaired in survivors of sepsis.

## Discussion

In our prospective, cross-sectional study we aimed to explore the phenomenon of sepsis-induced long-term immune paralysis. We identified patients who survived an episode of catecholamine-dependent sepsis 9–60 months prior to enrollment. General health status, immune cell subsets, cell surface receptor expression, HLA-DR expression, and cytokine secretion of stimulated whole blood were determined. We conducted our study to shed light on the phenomenon of persisting high morbidity in survivors of sepsis. In their review Winters et al. reported 1-year mortality after hospital discharge, which differed between 3 % and 43 % in 17 distinct studies [17]. In one other study, the 2-year mortality rate after hospital discharge was 44.9 % among individuals surviving severe sepsis. This was 1.5 times higher than the in-hospital mortality rate in the same study [20]. A very late increment for sepsis-related mortality was observed in a study that reported 1-year mortality of 51.4 %, increasing to 74.2 % 5 years after hospital discharge [21]. An impaired immune response as one sequelae of previous sepsis is believed to be a major contributing factor in these delayed deaths [16, 22]. To our knowledge studies investigating this phenomenon are rare, especially in humans. In distinct murine models, Marwart et al. demonstrated sepsis-induced loss of naive T cells but no prolonged defects in T cell function [23]. On analysis of myeloid cells after polymicrobial sepsis in mice, precursors of dendritic cells in the bone marrow were found to develop into regulatory dendritic cells that mediated immunosuppression [24]. Our own investigations showed that human sepsis induces distinct epigenetic changes in immunologically relevant genes that could ultimately contribute to functional changes in monocytes [25]. As these modifications are persistent and can be propagated, changes in basal regulatory mechanisms might also affect the immune function after surviving sepsis [22]. Additionally, effects on behavioral, affective and molecular responses in the brain of mice are shown after surviving LPS-induced systemic inflammation [26, 27].

There was almost complete matching of clinical characteristics that likely modify immune function (age, sex, presence of diabetes and renal function). As a result, the two groups were comparable in the context of the present study. The hypothesis of impaired immunity in survivors of sepsis is supported by the results of the clinical status questionnaire (Table 2). First, the observations revealed continuing infections even months and years after surviving sepsis. Second, the need for antibiotic drug prescriptions in four patients and two hospital admissions emphasize the severity and relevance of the infections. Third, we observed infections that are typically associated with impaired immune function: oral candidiasis, herpes zoster and lower airway infection. This is in line with mouse models, which indicate that survivors of septic shock are susceptible to infection with pathogens that are usually innocuous [24, 28–30]. Notably, these types of infection are likely to reduce the patient’s quality of life, an assumption that is consistent with previous reports describing impaired quality of life secondary to serious illness [17, 20].

The standard inflammatory markers were in the normal range or slightly increased, sufficient to exclude clinically relevant infectious episodes at the time of enrollment (Table 3). However, comparing the two study groups, the sepsis survivors had low CRP values, but higher CRP values than controls, and more frequently had slightly elevated PCT. This observation also supports the perception that sepsis is followed by a long-lasting, low-grade chronic inflammatory state.

Sepsis induces a comprehensive loss of myeloid cells and lymphocytes including CD4^+^ and CD8^+^ T cells [8]. Boomer et. al. observed a reduction in CD4^+^ and CD8^+^ T cells in the acute phase and an increase of regulatory T cells in the process of sepsis [31]. As shown by our own results the distribution of subsets seems to normalize in the long term after surviving sepsis (Fig. 1). Nevertheless, the presence of certain cells does not imply the exertion of function and several studies, mainly conducted in animals, show the presence of sustained functional alterations after severe infection [22, 28].

For the next investigation we therefore determined the expression levels of cell surface receptors on different T cell subsets (Fig. 2) and on monocytes (Fig. 3). In a longitudinal study, Boomer et. al. observed a trend in the MESF increase in PD-1 at the onset of sepsis [31]. In another study there was upregulation of PD-1 on T cells and PD-L1 on monocytes in patients with septic shock [32]. Relevant to the potential for clinical implementation, the blockade of PD-1 and CTLA-4 improves survival in primary and secondary fungal sepsis in mice [33]. In our study, we found contrary results for PD-1 in sepsis survivors in the long term (Fig. 2). However, BTLA receptor expression trended towards upregulation. As both are members of the important negative regulators of T cell function, clear-cut conclusions are hard to draw. It appears that the resulting phenotype might be highly dependent on the individual immune system, raising a question about the influential variables.

In our study, patients who survived an episode of sepsis had alterations in monocyte surface expression of pattern recognition receptors (Fig. 3). The most notable effect was in substantially decreased TLR5 expression. As TLR5 recognizes flagellin, which is the main component of bacterial flagella, the downregulation of TLR5 may in consequence contribute to impaired detection of certain bacteria by phagocytes.

Decreased surface HLA-DR expression on monocytes is a reliable surrogate marker of global immunosuppression and its value predicts mortality and development of nosocomial infection in patients suffering from sepsis [34–39]. As a reasonable consequence, survivors of sepsis in our study had no substantial differences in HLA-DR expression compared to controls (Fig. 4).

Boomer et al. have already showed that peripheral blood mononuclear cells isolated from whole blood 7 days after onset of sepsis have impaired secretion of IFN-γ when stimulated with α-CD3/28 [31]. According to our results, this trend appears to continue even months and years post-sepsis (Fig. 5). In contrast to our findings, the values for IL-6, IL-10 and TNF-α after α-CD3/28 stimulation were increased in that study [31]. As these patients were still hospitalized and in an acute immune condition, this could be one explanation for the difference. In another study from Boomer et. al., the cytokine secretions of TNF-α, IFN-γ, IL-6 and IL-10 were globally decreased after LPS and α-CD3/28 stimulation in patients who died from sepsis [15]. The same trends were observable in our group of sepsis survivors (Fig. 5).

Despite our valuable findings, there are several limitations in our study. First, our investigation is a pilot study and therefore we only examined a small number of patients. As a result we were only able to demonstrate pronounced trends and this is why our investigations need extensive revalidation with a larger sample size. Second, our control population consisted of mostly healthy individuals besides the described matching parameters. As so, we did not compare patients who had survived sepsis with patients who had survived non-septic critical illness. Therefore, our findings may be due to surviving an acute critical illness or non-matched co-morbidities and not necessarily from surviving sepsis. Third, we did not know the patients’ immune status before sepsis. The septic event could have evolved from previous differences in immune system function. These differences could be still detectable after sepsis, but not necessarily be a consequence of surviving sepsis. As most individuals had previously had urosepsis, different types of sepsis (e.g., abdominal sepsis) might have another fundamental effect on immune function and need to be evaluated. Furthermore, each individual was only investigated once and therefore, we were not able to identify possible changes in immune cell function over time.

We conducted this study as a basis for further investigation of a poorly studied group of patients: the survivors of sepsis. Even though long-lasting impairment of the immune system is believed to be the underlying reason for delayed sepsis-related death and to impact quality of life, studies investigating the immune status of survivors of sepsis in humans are missing. In the future, comprehensive studies need to test the correlation between the reasons (alterations of the immune cells) and the effects (quality of life and survival). Post-septic patients may require specific diagnostic tools and the results could enable us to identify patients who might benefit from structured post-sepsis care. Individualized medicine does not stop at the hospital door and therefore, we need to reconsider what comes after acute critical care.

## Conclusions

The reactivity of immune cells to different activators appears to be comprehensively impaired in survivors of sepsis. Among other reasons, this could be based on the downregulation of distinct cell surface receptors. Nevertheless, we believe that our findings might merely represent the tip of the iceberg, and further validation studies in larger cohorts are needed to clearly track the changes and the underlying molecular mechanism and their biological relevance.

## Key messages

- In the long term, survivors of sepsis had increased numbers of clinically apparent infections and a low-level inflammatory status based on the standard inflammatory markers
- In the post-sepsis group, there were alterations in monocyte surface expression of distinct pattern recognition receptors, most pronouncedly observed in decreased TLR5 expression
- Cytokine secretion in response to important activators of both the innate (LPS and zymosan) and the adaptive immune system (α-CD3/28) appeared to be weakened in sepsis survivors

## Abbreviations

ALT: alanine transaminase
AST: aspartate transaminase
BMI: body mass index
BTLA: B- and T-lymphocyte attenuator
CARS: compensatory anti-inflammatory response syndrome
CD: cluster of differentiation
CMV: cytomegalovirus
COPD: chronic obstructive pulmonary disease
CRP: C-reactive protein
CTLA: cytotoxic T-lymphocyte-associated protein
EDTA: ethylenediaminetetraacetic acid
FITC: fluorescein isothiocyanate
GGT: γ-glutamyl transferase
HIV: human immunodeficiency virus
HLA: human leukocyte antigen
ICD: International Statistical Classification of Diseases and Related Health Problems
ICU: intensive care unit
IFN: interferon
IL: interleukin
LPS: lipopolysaccharide
MCH: mean corpuscular hemoglobin
MCHC: mean corpuscular hemoglobin concentration
MCV: mean corpuscular volume
MESF: molecules of equivalent soluble fluorochrome
MODS: multiple organ dysfunction syndrome
PBMC: peripheral blood mononuclear cell
PBS: phosphate-buffered saline
PCT: procalcitonin
PD: programmed cell death
PICS: persistent inflammation-immunosuppression catabolism syndrome
SIRS: systemic inflammatory response syndrome
TLR: toll-like receptor
TNF: tumor necrosis factor
WBC: white blood cell count
